# The Book World of Medicine and Science

**Published:** 1899-06-24

**Authors:** 


### The Book World of Medicine and Science.

Constipation and its Modern Treatment. By George Herschell, M.D.Lond. (London: H. J. Glaisher. 1899. Pp. 67. Price 2s. 6d. net.)

This little book is a reprint of a paper read by Dr. Herschell before the West Kent Medico-Chirurgical Society in 1898. The subject is, no doubt, trite enough; most practitioners are familiar with the rational treatment of constipation, though their patients still prefer cathartic "liver pills," and even have a certain pride in the amount of aperient drugs they can consume without result. Still, Dr. Herschell has succeeded in investing his subject with fresh interest inasmuch as he has not attempted to deal with every aspect of the question, but has, in the main, contented himself with drawing attention to certain factors in the causation of constipation, and to certain methods of treatment which are not, perhaps, so generally known as they should be. Dr. Herschell advises, of course, in the first place a careful attempt to determine the exact cause and nature of the constipation, and is accustomed to make use of Gersuny's valuable sign, and of Illoway's ingenious application of a soda water syphon to the exigencies of the consulting-room. In discussing treatment, Dr. Herschell liberally gives practical "tips"; the little "dodges" of practice—such as the use of olive oil and ox-gall as a fæcal solvent—which mean so much to the general practitioner; and he devotes no inconsiderable amount of space to the description of methods of restoring tone to the abdominal muscles by electrical "vibration." It would be idle to comment on the omissions from Dr. Herschell's book, for, as we have said, we take it he intended merely to lay stress on certain points to which he has devoted particular attention. And these are points with which every practitioner should be familiar.

Materia Medica and Therapeutics. By J. Mitchell Bruce, M.A., M.D., &c. (London: Cassell and Co., Limited. 1899. Pp. 606. New Edition. 38th thousand. Price 7s. 6d.)

The familiar red-edged and red-covered book—known affectionately by all students as "Mitchell Bruce"—has, in the present issue, been thoroughly revised and adapted to the new British Pharmacopœia. A book such as this—in its way a classic—calls for no adverse criticism and stands in no need of favourable notice. But we may be allowed to point out that this work has been considerably enlarged, and that, as its accomplished author states, greater detail has been introduced respecting the chemical and pharmaceutical relations of individual drugs. The only fault to be found in the work is a fault for which the author is not, of course, responsible: the fault of the British Pharmacopœia in its resolute adherence to the absurdity of obsolete weights and measures. Reading Dr. Bruce's work over again we are anew impressed with the lucidity—and, if we may be allowed the term, the sanity—of the second part of the work—those chapters on "General Therapeutics," which are, we fear, unduly neglected by students with an eye to requirements of the examining boards. We do not wish to be frivolous, but we recall an incident that occurred at one of Dr. Bruce's post-graduate lectures. Dr. Bruce had lectured with his usual brilliancy on "Cough," and had specially insisted on the value of careful investigation and accurate analysis of signs and symptoms before determining treatment. At the close of the lecture an American visitor went up to the speaker and said, "Say, now, Doctor, it would be real elegant if you gave us a prescription for cough!" In all seriousness the clinical vice of our American colleague is a very common one, and we can think of no better corrective than an intelligent study of Dr. Bruce's writings.

Milk: Its Nature and Composition. A Handbook on the Chemistry and Bacteriology of Milk, Butter, and Cheese. By C. M. Aikman, M.A., D.Sc. (London: Adam and Charles Black. 1899. Pp. 178. Second Edition.)

This is a well-intentioned little book, written apparently with the praiseworthy purpose of affording accurate information to the students of "agricultural science." It deals pleasantly, lucidly, but, we are sorry to say, superficially, with a large number of interesting and difficult problems. We cannot truthfully say that it is likely to do much more than to awake interest in the matters with which it deals. For example, the matters of sterilisation and Pasteurisation are treated of. Yet no practical details are given, no steriliser is described or figured. And while the author expresses the hope that the day is not far distant when only sterilised or Pasteurised milk will be used, he gives no warning of the now familiar evils resulting from its use. Indeed, he says it is especially for children that sterilised milk should be employed. We are told that in this second edition the text has been thoroughly revised. We perforce must then call attention to some serious misstatements. On page 103 we are told—and the error is not that of the printer—that the typhus bacillus has been found in milk, and that a number of typhus epidemics have been traced to this source! Further on we read that the microbes causing scarlet fever and diphtheria have not yet been isolated! On page 77 the familiar sporulating "drumstick" bacilli of tetanus are named "spores." Careless inaccuracies of this nature render a book of little value, and perhaps it is as well that the author does not venture to describe in any detail the bacteriological methods now employed in Dutch or Danish dairies.

